# Comment On “Main pancreatic duct exposure, repair or reconstruction during minimally invasive pancreatic enucleation: long-term metabolic outcome from a prospective cohort study (CSPAC-MIEN-1)*”*

**DOI:** 10.1097/JS9.0000000000003191

**Published:** 2025-08-22

**Authors:** Qun Xiang, Yan Ma, Jingjing Wang

**Affiliations:** a Department of Infectious Diseases, Hainan Public Health Clinical Center, Haikou, Hainan Province, China; b Department of Gastrointestinal surgery, Hainan Affiliated Hospital of Hainan Medical University, Haikou, Hainan Province, China

*Dear Editor,*

We read with great interest the article titled “Main pancreatic duct exposure, repair or reconstruction during minimally invasive pancreatic enucleation: long-term metabolic outcome from a prospective cohort study (CSPAC-MIEN-1)^[1]^.” This study provides valuable insights into the long-term metabolic outcomes of minimally invasive pancreatic enucleation with MPD exposure, repair, or reconstruction (ERR), and we commend the authors for their significant contribution to the field. However, we would like to address a concern that may impact the completeness and generalizability of the findings. This manuscript was developed following the TITAN Guidelines 2025^[2]^ for the declaration and use of artificial intelligence in scholarly publishing . No AI tools were employed in the conception, design, analysis, interpretation, or writing of this work.

While the authors have thoroughly analyzed the outcomes of MPD ERR, the study does not sufficiently address the potential influence of tumor type distribution on the necessity and success of MPD repair. The results indicate a significantly higher proportion of cystic tumors in the MPD ERR group (58.1%) compared to the MPD non-exposed group (35.0%, P < 0.001, Table 1). Given that cystic tumors are more likely to encase or compress the MPD, this may increase the need for MPD repair or reconstruction, potentially skewing the outcomes associated with this procedure.

Previous studies have shown that cystic tumors, particularly those that encase or compress the MPD, present distinct challenges in pancreatic surgery and may lead to higher rates of complications^[3]^. These factors could influence both the perioperative and long-term metabolic outcomes in ways that are not fully captured in the study’s current design.

We recommend that future studies consider stratifying the results based on tumor type to better understand how tumor characteristics affect the need for MPD ERR and the associated outcomes. This would provide a clearer understanding of the impact of tumor characteristics on the procedure and help avoid confounding factors in the analysis of MPD-related complications and long-term metabolic function.

## Data Availability

The letter does not have any data.

## Research registration unique identifying number (UIN)

Not registered for the type of the study.
